# Transcranial Direct Current Stimulation in Older Adolescent with Chronic Mild Traumatic Brain Injury: A Case Study of Clinical and Functional Connectivity

**DOI:** 10.21203/rs.3.rs-8058808/v1

**Published:** 2025-11-18

**Authors:** Michelle Eliason, Elizabeth Castro, Elizabeth Fonfara, Ghazala T. Saleem

**Affiliations:** Brain Function and Recovery Lab, Rehabilitation Sciences, University at Buffalo, Buffalo, NY, United States.; Neuroscience Program and UBMD Orthopaedics and Sports Medicine, Department of Orthopaedics, University at Buffalo, NY, United States.; Department of Biomedical Engineering, University at Buffalo, NY, United States; Director, Brain Function and Recovery Lab, Rehabilitation Sciences, University at Buffalo, Buffalo, NY, United States.

**Keywords:** Traumatic Brain Injury, Adolescent Brain Injury, Transcranial Direct Current Stimulation Case Study

## Abstract

This is the first case study, to our knowledge, to document pre- and post-tDCS resting-state functional MRI changes between the dorsolateral prefrontal cortex (DLPFC) and brainstem in an adolescent with chronic post-concussive symptoms following multiple mild traumatic brain injuries (mTBIs). By demonstrating that cortico–brainstem connectivity can be modulated through noninvasive neuromodulation, the novel findings of this case study attempt to inform mTBI treatment in adolescents exhibiting residual post-mTBI symptoms. A 19-year-old female with a history of three mTBIs (most recent five years prior) and persistent post-mTBI depression, anxiety, and post-concussive symptoms exhibited severe baseline hypoconnectivity between DLPFC and subcortical–brainstem nuclei, including the pontine reticular formation, raphe nuclei, and substantia nigra. Baseline behavioral testing revealed severe depressive and anxiety symptoms and elevated concussion symptom burden. The patient received transcranial direct current stimulation (tDCS; 1.5mA, 20 min, twice weekly for five weeks) with an anode over the left DLPFC and cathode over the contralateral supraorbital area. Post-intervention imaging revealed widespread polarity reversals and increased connectivity between DLPFC and brainstem arousal nuclei (e.g., periaqueductal gray, pontine reticular formation), alongside continued hypoconnectivity in serotonergic and dopaminergic nuclei. Clinically, depressive symptoms improved modestly, while anxiety and concussion symptom burden increased. This case demonstrates that adolescent mTBI can produce long-lasting disruption in prefrontal–brainstem networks and that tDCS may rapidly modulate these circuits in both restorative and deregulatory directions. Notably, neuromodulation may amplify arousal systems before stabilizing cortical control, underscoring the need for individualized montage design, close clinical monitoring, and longitudinal follow-up in post-mTBI rehabilitation.

## Introduction

Although brain injury severity ranges from mild to severe [1], mild TBI (mTBI) is most prevalent [2], and can lead to participation barriers [2] and short- and long-term comorbidities like headaches, fatigue, sleep disturbances, mood disturbances, and post-traumatic epilepsy [3–5]. Among adolescents, those aged 15–19 years are most likely to require emergency department care or hospitalization for head injuries[6, 7] [5], with sports accounting for over 40% of all mild Traumatic Brain Injuries (mTBIs) [2]. Youth who sustain a Traumatic Brain Injury (TBI) later, in grades 9–12 (ages 14–19), demonstrate poorer academic performance [8] and greater medical utilization than those injured during younger years [2]. Longitudinal data show that children with mTBI exhibit persistent deficits in attention, hyperactivity-impulsivity, information processing stability, and visual working memory, resulting in academic declines in technical reading performance up to two years post-injury [4]. Collectively, these findings underscore that pediatric and adolescent mTBI can produce enduring neurodevelopmental, behavioral, and academic-related barriers, emphasizing the need for early detection, improved diagnostic criteria, and long-term monitoring of this disorder [4]. Importantly, mounting evidence indicates that mTBI represents a chronic neurological condition rather than an isolated event, with persistent structural and functional alterations that may continue to evolve long after the initial insult [9–12]. This chronic trajectory underscores the need for continued investigation into neural mechanisms of recovery and long-term modulation.

Symptoms experienced after an mTBI are theorized to arise from various cortical and subcortical areas (e.g., damage to the brain stem) [13–15]. Imaging studies show that cortical–subcortical interactions, particularly between the dorsolateral prefrontal cortex (DLPFC), thalamus, and anterior cingulate cortex (ACC), are disrupted after TBI, contributing to both cognitive and behavioral symptoms [13]. These disruptions highlight the importance of examining deeper brain structures involved in information integration and regulation. Among these, the brainstem is a critical hub linking cortical and subcortical networks essential for maintaining motor, sensory, and cognitive function [16]. The brain stem connects the cerebrum, cerebellum, and spinal cord and contains nuclei that originate several neurotransmitter systems modulating cortical excitability and arousal [17] [18]. Given its dense architecture and integrative role in sensorimotor and cognitive regulation, the brainstem is particularly vulnerable to shearing and diffuse axonal injury even after mild insult. Emerging neuroimaging evidence shows that mTBI can cause structural alterations in brainstem regions, particularly the pontine reticular formation, associated with executive dysfunction, working-memory impairment, and slowed processing speed [19, 20].

Historically, most of what is known about brainstem function comes from animal studies focused on brainstem nuclei or pathways [18], leaving gaps in understanding how these structures interact with cortical regions. However, the development of high-resolution brainstem atlases and resting-state functional connectivity (rs-FC) mapping now enables visualization of brainstem-cortical communication in humans [18]. Recent findings show robust rs-FC between the brainstem and regions implicated in motor control, cognitive regulation, and neurotransmission [18, 21, 22]. Damage to midline subcortical structures, including the thalamus, midbrain, and middle cingulate cortex (MCC), has been shown to disrupt large-scale networks including the default mode network (DMN) and DLPFC circuits governing executive and emotional regulation [14, 15]. Although the brainstem is anatomically and functionally distinct from these subcortical regions, it maintains extensive connections with cortical and limbic areas involved in attention, arousal, and emotional control [18, 23–26]. Brainstem nuclei further regulate neurotransmission, vigilance, and autonomic tone [18, 22]. Examining rs-FC between the regions of the DLPFC and brainstem may elucidate the cognitive, emotional, and behavioral sequelae observed in adolescents with mTBI [27].

DLPFC–brainstem dysconnectivity is associated with post-concussive fatigue, mood disturbance, and impaired cognitive flexibility [28–30], making prefrontal–brainstem connectivity a mechanistic marker of emotional regulation and recovery following neuromodulation. Furthermore, pediatric imaging studies rarely assess the combined cortical (e.g., DLPFC, supplementary motor area) and subcortical (e.g., thalamus, brainstem) alterations that jointly mediate attention, executive control, affect, and arousal regulation following injury [15, 19].

Noninvasive brain stimulation approaches, such as transcranial direct current stimulation (tDCS), have emerged as promising tools to modulate cortical excitability and functional network organization [31–33]. In adolescents, tDCS has been shown to safely engage prefrontal circuitry associated with cognitive and emotional regulation [34]. While traditional adult models describe polarity-dependent (anodal vs. cathodal) excitatory and inhibitory effects, developmental evidence suggests that the neuromodulatory response may be more variable and state-dependent, reflecting broader shifts in oscillatory and network dynamics rather than fixed polarity effects [35, 36]. Variable and state-dependent neuromodulatory effects call for individualized, mechanism-informed approaches to understand how tDCS influences brain–behavior relationships in youth recovering from mTBI. Here, we aim to examine the impact of prefrontal transcranial direct current stimulation (tDCS) on rs-FC between frontal cortical regions and brainstem nuclei in an adolescent with persistent post-mTBI symptoms, with attention to changes in depression- and anxiety-related symptoms.

### Case Study Methods

In this case study, one experimental participant (hereafter referred to as ‘participant’) with persistent post-concussive symptoms following mTBI and ten healthy controls (ages 10–21) were enrolled under IRB approval with informed consent and assent. All completed baseline MRI and behavioral assessments. The participant then underwent tDCS, using a Neurocom (Germany) DC stimulator with two 5×7 electrodes. Anode placement was over the left DLPFC and cathode over the contralateral supraorbital space. Active tDCS stimulation occurred twice weekly for five weeks (1.5mA × 20 min, 30 s ramp up), followed by repeat MRI and behavioral testing. A sham condition was not included because the participant elected to discontinue participation after the active tDCS phase, precluding the possibility of adding a subsequent sham condition. Inclusion criteria required English proficiency and no neurological, developmental, or orthopedic disorders; exclusions included loss of consciousness > 30 min, posttraumatic amnesia > 24 h, abnormal neuroimaging, seizure history, or MRI contraindications.

To complement our stimulation site, we examined rs-FC in three adjacent regions: Brodmann area 9/46 (core DLPFC), area 44 (inferior frontal gyrus/Broca’s region), and area 8 (frontal eye field). These areas were selected a priori due to their proximity to the stimulation target and their respective roles in executive function, cognitive control, and top-down modulation of brainstem and limbic circuits [37]. Regions of interest included dorsal raphe (DR), inferior olivary nucleus (ION), median raphe (MR), periaqueductal grey (PAG), pontine reticular formation (PRF), nucleus raphe magnus (NRM), red nucleus subregions 1(RN1) and 2 (RN2), and substantia nigra (Pars Reticula [SNr] and Pars Compacta [SNc]) [18, 22](Table 1).

### Imaging Acquisition Parameters

Imaging was performed on a 3T Philips MR 7700 at an academically-affiliated Center for Biomedical Imaging using a 32-channel head coil. Sequences included T1-weighted, T2-weighted, and FLAIR acquisitions. All scans were reviewed by a pediatric neuroradiologist and medical physicist for incidental findings. Any clinically significant findings were communicated to the occupational therapist and neuroscientist for appropriate follow-up with the participant or guardian.

rs-FC data were acquired using a Human Connectome Project–like blood-oxygen-level dependent (BOLD) single-shot, partially parallel gradient-recalled echo-planar sequence with sensitivity encoding (SENSE). Parameters were: repetition time (TR) = 800ms, echo time (TE) = 37ms, flip angle = 52°, SENSE acceleration factor = 1.15, 2-mm axial slices with no gap, and in-plane resolution = 2 × 2 mm (104 × 103 voxels). A magnetization-prepared rapid gradient echo (MPRAGE) scan (1 mm^3^ isotropic voxel; TR/TE/TI = 8.2/3.7/900ms) was obtained for registration.

### Patient Information

A 19-year-old female with a history of three prior mTBIs sustained between the ages of 9 and 13 presented for evaluation. The most recent injury occurred in October 2019, approximately five years before participation in this study. Two of the injuries were sustained during competitive gymnastics, and the third resulted from a fall from a bed. The participant denied loss of consciousness exceeding 30 minutes or post-traumatic amnesia lasting more than 24 hours but was clinically diagnosed with an mTBI following each event.

After the first injury, the participant was temporarily restricted from returning to gymnastics until symptoms subsided; however, she reported that her symptoms were never fully resolved. During this four-year period of recurrent concussions, she was diagnosed with major depressive and generalized anxiety disorders, common comorbidities after sustaining an mTBI[3–5] and chronic migraines/headaches. She denied current dizziness or visual disturbances. The participant’s mother reported noticeable personality changes emerging in the participant following the mTBIs including increased social isolation, avoidance of social activities she once enjoyed, and significant anxiety and depressive symptoms beyond that. She further reported that because pharmacological interventions and counseling provided minimal improvement, her daughter’s optimism about treatment effectiveness markedly declined.

### Timeline

**Figure 1:**
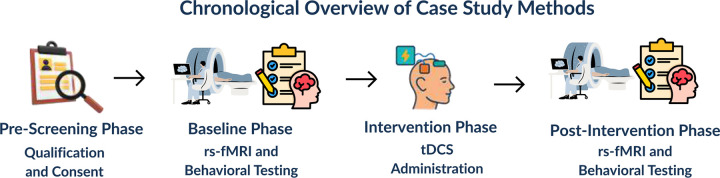
Chronological overview of the case study timeline and data collection phases. The *Pre-Screening Phase* (qualification and consent) was followed by the *Baseline Phase (T1)*, during which resting-state functional MRI (rs-fMRI) and behavioral testing were conducted to establish pre-intervention measures. The *Intervention Phase (T2)* involved tDCS administration. The *Post-Intervention Phase (T3)* included repeated rs-fMRI and behavioral assessments to evaluate post-stimulation.

The study followed a sequential protocol of pre-screening, baseline assessment, neuroimaging, neuromodulation, and post-testing (Figure 1). During pre-screening, the participant completed eligibility, safety, and consent forms, including functional magnetic resonance imaging (fMRI) prescreening, incidental findings disclosure, eligibility and medical history questionnaires, and post-consent pregnancy screening for females ≥14 years.

At baseline, behavioral testing evaluated cognitive, affective, and motor domains using the primary and secondary measures. Primary endpoints include Patient Health Questionnaire–9 (PHQ-9) [38, 39], Generalized Anxiety Disorder–7 (GAD-7) [40], Wechsler Abbreviated Scale of Intelligence–Second Edition (WASI-II)[41], and Post-Concussion Symptom Scale (PCSS) [42] to quantify depression, anxiety, verbal fluency, and post-concussive symptoms (Table 2). This study will only analyze primary outcome measures. Secondary endpoints include Demographic and Medical History Questionnaire, Maternal History Questionnaire, Physical and Neurological Examination for Subtle Signs (PANESS)[43], Nine-Hole Peg Test (NHPT)[44], Dual Tasking Screen [45], and computerized Posturography, An rs-fMRI scan at an academically-affiliated imaging center provided a neurophysiological baseline. The participant then received tDCS using a unihemispheric montage (anodal = target; cathodal = reference) [46].

Immediately post-tDCS, a second rs-fMRI scan with identical acquisition parameters enabled within-subject comparison of pre- and post-stimulation connectivity. Post-testing repeated the all of the same primary and secondary measures taken at baseline to assess short-term changes in mood, anxiety, and concussion-related symptoms. This organization ensured transparency, reproducibility, and compliance with CARE case-reporting standards for neurorehabilitation and neuromodulation research.

### Primary Behavioral Testing

#### PHQ-9:

The PHQ-9 is a 9-question self-reported measure and was used to determine the level of depressive symptoms the participant was experiencing. Scores of 1–4 indicate minimal depression, 5–9 mild depression, 10–14 moderate depression, 15–19 moderately severe depression, and 20–27 severe depression [38, 39].

#### GAD-7:

The GAD-7 is a 7-question self-reported scale used to assess the severity of anxiety symptoms. Scores of 0–4 indicate minimal anxiety, 5–9 mild anxiety, 10–14 moderate anxiety, and 15–21 severe anxiety [40].

#### PCSS:

The PCSS is a 21-question self-reported measure used to quantify concussion-related symptoms. Higher total scores reflect a greater number and severity of post-concussive symptoms [42].

#### WASI-II:

The WASII-II is a brief, four-subtest measure of intellectual functioning (Vocabulary, Similarities, Block Design, Matrix Reasoning) that yields Verbal Comprehension, Perceptual Reasoning, and Full-Scale IQ scores). It provides a reliable estimate of global cognitive ability consistent with crystallized and fluid intelligence theory and demonstrates sensitivity to age-related and neurological differences in cognitive performance [47, 48]

### Image Processing

Connectivity analyses were derived from the Eagle Atlas, which integrates seven parcellations (A1–A3, B1–B4; total 459 ROIs). Only three sub-atlases contained regions relevant to this study (A2, B2, B3). From these, 26 ROIs were initially selected: eight frontal (A2), sixteen brainstem (B2), and two subthalamic (B3). Because the primary analysis targeted dorsolateral prefrontal–brainstem interactions, only the six DLPFC ROIs from A2 and sixteen brainstem ROIs from B2 were retained, yielding 6 × 16 = 96 possible ROI–ROI connections. Four ROIs (two premotor, two subthalamic) were excluded prior to analysis[49].

Data were converted from DICOM to NIfTI in BIDS format using *BIDS’em ALL* [50].

Preprocessing and rs-FC analyses were conducted in the CONN Functional Connectivity Toolbox [51] [52]. Steps included functional realignment and unwarp, slice-timing correction, outlier identification, direct segmentation and normalization, and 8-mm Gaussian functional smoothing [52]. Denoising procedures removed non-neural signal components using linear regression and temporal band-pass filtering (0.01–0.1 Hz).

Since only one concussed participant was available, first-level analysis was performed to generate individual resting-state correlation matrices. T1-weighted and fMRI data were registered, and the Eagle atlas (integrating the Brainnetome [a2][53], Brainstem [b2][54], and Diencephalon [b3] [54]atlases) [55] was applied to define regions of interest (ROIs): DLPFC, premotor cortex, reticular formation, substantia nigra, subthalamic nucleus, RN, ION, NRM, MR, DR, and PAG.

ROI-to-ROI connectivity (RRC) analyses quantified resting-state functional connectivity (rs-FC) across all ROI pairs using weighted least-squares linear modeling [52]. Because second-level (group) analysis was not possible, individual RRC matrices were exported to text files and filtered to retain only 96 connections between prefrontal and brainstem regions, which were the focus of this study.

### Statistical Analysis

#### Imaging:

To assess deviation from the control sample (n = 10), z-scores were calculated for each connection as:

Eq. 1 $$z\;\text{score}=\frac{\left(\text{Participant}\;\text{correlation}\;\text{coefficient}-\text{Control}\;\text{mean}\right)}{\text{Control}\;\text{standard}\;\text{deviation}}$$

Each of the 96 connections produced a pre-tDCS and post-tDCS z-score. Values with |z| ≥ 2 were considered statistically abnormal, representing either higher (z > 2) or lower (z < −2) resting-state functional connectivity (rs-FC) relative to controls [56].

All descriptive and correlation analyses were conducted using R (version 2025.09.1+401).

#### Descriptive Analysis:

Data were screened and preprocessed to ensure proper formatting and variable integrity. The *Group* column was parsed to separate “TBI_pre” and “TBI_post” entries into distinct Group and Timepoint variables, enabling within-subject longitudinal comparison for the case participant. Controls were analyzed as a single group to establish normative descriptive statistics. For the control cohort, descriptive statistics were computed for each variable, including the mean, standard deviation, median, and interquartile range (IQR). These summaries provided normative reference values for subsequent single-case z-score contextualization. For the participant, pre- and post-intervention data were isolated and ordered chronologically. Within-subject change scores were calculated for each measure by subtracting pre-intervention values from post-intervention values (Table 2).

#### Fisher z-transformed Correlation Analysis:

**Figure 2:**
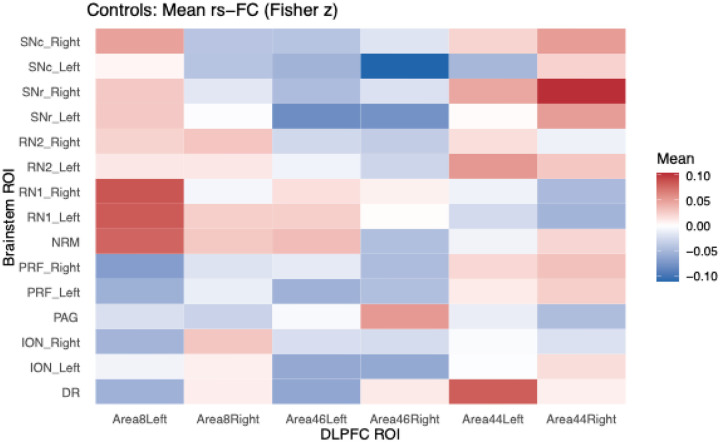
Mean resting-state functional connectivity (Fisher z) among control participants, showing normative patterns of DLPFC–brainstem coupling used as the reference for participant comparisons.

**Figure 3:**
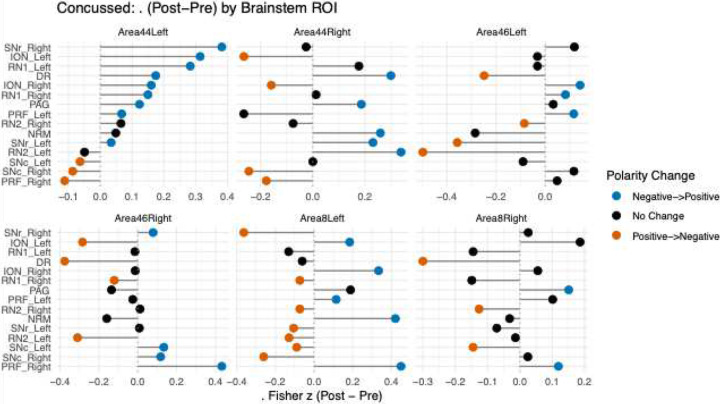
illustrates prefrontal–brainstem connectivity changes (Δ Fisher z) from pre- to post-tDCS for single TBI participant across all cortical–brainstem pairs, where positive values indicate increased and negative values indicate decreased functional connectivity relative to baseline. Colored points indicate edges that reversed correlation from pre- to post-tDCS (blue = negative→positive; orange = positive→negative), highlighting directional shifts in cortico–brainstem coupling.

We examined cortico–brainstem connectivity between DLPFC subregions (e.g., Area 8, 46, and 44, left/right) and brainstem/arousal nuclei. All statistical analyses were conducted on Fisher z–transformed correlation coefficients per edge (DLPFC ROI × brainstem ROI) for 10 controls and one mTBI case (pre/post) [57]. For each edge, we computed control mean and SD across the 10 controls. Distributions were visualized with DLPFC × brainstem heat map summarized control mean rs-FC to contextualize network topology (Figure 2). For each edge, we calculated the case participant’s change Post → Pre. We classified correlation sign reversals when the Fisher-z sign crossed zero (positive→negative or negative→positive) and summarized those edges in a lollipop plot (Figure 3). Because the control sample included participants with age variability (11–21 years), we applied age-adjustment to ensure that observed deviations reflected connectivity differences rather than developmental effects. For each connection, the relationship between age and connectivity was modeled across controls, and the expected value for age 19 (the participant’s age) was estimated from this regression (connectivity ~ age). The age-adjusted z-score was then calculated as:

Eq. 2 $$z_{\text{age-adjusted}}=\frac{\left(\text{Participant}\;\text{value}-{\hat{y}}_{\text{age}=19}\right)}{\text{Standard}\;\text{Deviation}\;\left(\text{SD}\right)\;\text{of}\;\text{residuals}}$$

where *ŷ*_age=19_ represents the predicted connectivity at age 19 from the control regression model, and the denominator (SD of residuals) reflects variability unexplained by age. This approach enables comparison of the participant’s data to the age-expected connectivity pattern derived from the control group. Edges exceeding |z| ≥ 2 at pre, post, or change were flagged as atypical.

## Results

### Behavioral testing

The participant with TBI was 19 years old at the time of assessment pre and post intervention (Table 2). For reference, the comparison sample (n = 10) had a mean age of 17.8 years (SD = 3.55), with average scores of 4.9 (SD = 6.08) on the PCSS, 3.5 (SD = 3.81) on the GAD-7, and 4.2 (SD = 2.82) on the PHQ-9. WASI-II subtest means were 15.6 (SD = 4.62) for Matrix Reasoning and 38.4 (SD = 4.88) for Vocabulary (Table 2).

### Control rs-FC Metrics

Across ROIs, baseline mean connectivity values were relatively low in magnitude, with some regional variation. For Area 44 (left hemisphere), connectivity to the DR, ION, NRM, and PAG showed small absolute means (e.g., DR: M = 0.083, SD = 0.139; PAG: M = −0.012, SD = 0.131)(See Table 1). Area 46 showed similar modest mean correlations to subcortical regions across hemispheres. Notably, left Area 44→right SNr yielded a positive mean connectivity of 0.046 (SD = 0.132), whereas right Area 46→left SNc showed a mild negative mean of −0.110 (SD = 0.118). In general, connectivity estimates were close to zero, but multiple regions (e.g., Area8→RN1, Area44→PRF) demonstrated consistent laterality differences and notable interhemispheric variability(Figure 2).

Across all dorsolateral prefrontal–brainstem connections, 53 correlation reversals (sign changes in Fisher z connectivity) were observed from pre- to post-tDCS. The largest negative to positive shifts occurred between Area 44 left and SNr right (**Δ** = 0.382), ION left (Δ = 0.314), and RN1 left (**Δ** = 0.283), reflecting strengthened connectivity relative to baseline. The most prominent positive to negative reversals appeared in Area 46 left–RN2 left (**Δ** = −0.498), Area 46 right–DR (**Δ** = −0.376), and Area 8 left–SNr right (**Δ** = −0.364), indicating reduced coupling after stimulation. Correlation reversals were distributed across bilateral DLPFC regions, suggesting heterogeneous directionality of modulation within cortico–brainstem pathways rather than uniform facilitation or suppression (Table 2, Figure 3).

### Correlation Changes

#### Large-magnitude changes (e.g., |z| ≥ 2)

**Figure 4:**
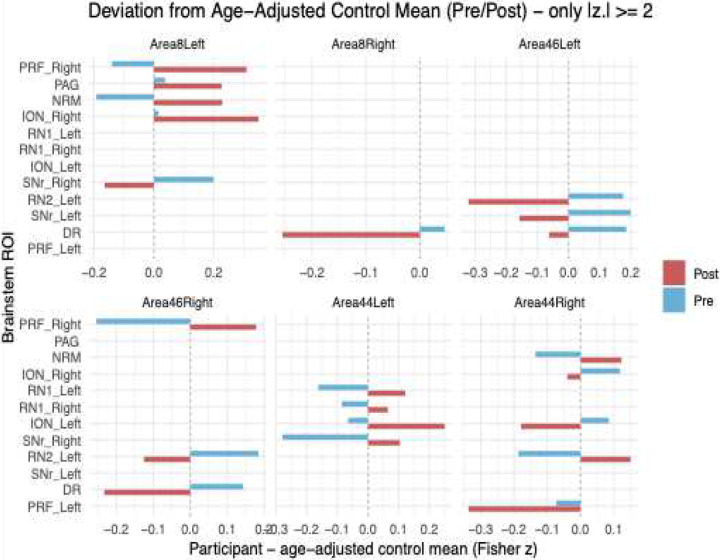
Age-adjusted deviations in resting-state functional connectivity (rs-FC) between dorsolateral prefrontal cortex (DLPFC) and brainstem regions for the concussed participant relative to controls. Bars represent z-scored deviations from the age-predicted control mean (|zΔ| ≥ 2), with positive values indicating stronger and negative values indicating weaker connectivity compared to age-adjusted norms.

Following the intervention, several cortical–brainstem ROI pairs exhibited large-magnitude changes in Fisher z-transformed connectivity values (defined here as |Δz| ≥ 0.25) when compared against age-adjusted control means. Notably, Area 44 left showed marked increases in connectivity with the ipsilateral ION (Δz = +0.314), the left RN1 (Δz = +0.283), the right RN1 (Δz = +0.150), and the right SNr (Δz = +0.382). In contrast, Area 44 right demonstrated mixed changes, including decreases in connectivity with both the left (Δz = −0.264) and right (Δz = – 0.159) ION, and the left PRF (Δz = −0.264), alongside increases with the NRM (Δz = +0.259) and left RN2 (Δz = +0.338). Area 46 left showed notable reductions in connectivity with the DR (Δz = −0.248), Left RN2 (Δz = – 0.498), and left SNr (Δz = −0.358). Similarly, Area 46 right demonstrated a decrease in connectivity with the DR (Δz = −0.376). Area 8 left was associated with substantial post-intervention increases in connectivity with the right ION (Δz = +0.334), NRM (Δz = +0.421), PAG (Δz = +0.189), and right PRF (Δz = +0.449). A modest reduction was also observed in connectivity with Left SNr (Δz = −0.106). Finally, Area 8 right demonstrated a notable decrease in connectivity with the DR (Δz = −0.300) (Table 2, Figure 4).

##### Outliers:

Links involving the median raphe exhibited extreme outliers post-tDCS. For clarity in case study figures, these points were masked to 0 for visualization. All summary statistics and thresholds were derived from the original values.

## Discussion

### TBI Participant Perspective

The participant tolerated all stimulation sessions well without complaints of pain, though she described mild tingling and itching sensations during 4 of the 10 sessions. These sensations were transient and did not interfere with participation. When informally asked throughout the intervention period whether she perceived any positive changes in her daily functioning or mood, the participant consistently reported no noticeable improvements. During the final session of active tDCS, the participant elected to withdraw from the study, resulting in an attrition rate of 100%. She expressed that her symptoms had intensified, describing increased anxiety and fear that made her reluctant to leave her home. She reported increased internal narrative of anxiety-related topics. Despite this subjective worsening, she remained compliant with all study procedures and completed all scheduled sessions prior to withdrawal. These concerns were not disclosed during post-session debriefings, suggesting a possible underreporting of symptom distress during the intervention period. At the final visit, the participant was counseled to report any ongoing or worsening symptoms to her medical provider and to seek local mental-health counseling for mood exacerbations. She denied suicidal ideation, intent, or thoughts of self-harm.

### Behavioral Analysis

Brain physiology is altered at baseline in older children after mTBI compared to healthy controls. Behavioral assessments revealed striking differences: the experimental participant scored 22 on the Patient Health Questionnaire (PHQ-9) at baseline, indicating severe depression, compared to a control group average of 4.2 (minimal depression). Similar elevations were noted on GAD-7 (17 vs. 3.5) and PCSS (39 vs. 4.9). At the neural level, the participant exhibited profound hypoconnectivity between DLPFC and key subcortical/brainstem structures at baseline, including the PRF and RN, with several connections showing z-scores below −3.0 (e.g., Area *8* → *PRF Left: z = −3.60*). This pattern suggests under-recruitment of regulatory networks involved in arousal, motor control, and affective modulation, possibly contributing to the participant’s elevated symptom burden prior to intervention

Despite a slight improvement from 22 to 19 on PHQ-9, both GAD-7 (17 to 20) and PCSS (39 to 46) worsened, aligning with the participant’s report of increased internal distress and functional withdrawal. Neuroimaging revealed hyperconnectivity from Area 8 and Area 44 (Left and Right) to the PAG and brainstem arousal nuclei like PRF, alongside persistent hypoconnectivity of regulatory outputs (e.g., Area 9/46). This pattern may reflect heightened arousal network engagement without compensatory prefrontal inhibition, consistent with physiological hyperarousal rather than clinical improvement.

This divergence between subjective symptom amplification and objective connectivity shifts highlights how neuromodulation can initially destabilize interoceptive and vigilance systems, particularly in individuals with pre-existing affective sensitivity. The participant’s full procedural compliance followed by withdrawal from the study underscores the gap between tolerability and emotional safety. Mild paresthesia were reported in 4 of 10 sessions but were transient and did not interfere with participation. Her decision to withdraw was instead linked to increased anxiety, fear, and negative internal narrative. From a mechanistic standpoint, stimulating frontal control networks with tDCS may, in some individuals, temporarily amplify worry and perseverative thinking [58].

Our montage (anode: left DLPFC; cathode: right OFC) likely increased excitability in cognitive control circuits (Area 9/46), while simultaneously reducing right OFC-mediated inhibition of limbic-brainstem arousal systems [59]. Forehead cathode placement (supraorbital) may have also co-stimulated the ophthalmic (V1) branch of the trigeminal nerve, which has bidirectional connections with the locus coeruleus and can modulate arousal [60–64]. Because scalp current can shunt through low-resistance tissues, cephalic forehead returns also reduce cortical specificity [65]. The selected stimulation intensity (1.5 mA for 20 minutes) reflected a conservative yet sufficient dose within the current adolescent tDCS literature, where recommended parameters typically range between 0.5 and 2 mA [66–68]. This level was chosen to balance safety and cortical engagement given the absence of standardized best practices for youth populations. While lower intensities may yield more variable or delayed neuromodulatory responses, higher amplitudes could theoretically amplify excitatory–inhibitory network shifts [35, 36]. Consistent with these mechanisms, post-tDCS data showed increased coupling between prefrontal regions (Area 8, 44) and arousal-promoting nuclei (PAG, PRF), alongside connectivity shifts involving raphe nuclei (Table 2). This aligns with the participant’s subjective experience of rumination and emotional intensification. Notably, Area 44 (part of Broca’s area) showed increased connectivity with both dorsal raphe (bilaterally) and nucleus raphe magnus (right), which may reflect greater integration between language circuits and serotonergic mood regulation, potentially explaining the 7-point gain in WASI-II Verbal Comprehension. Because this is a single-case design, findings cannot infer causality. However, this case illustrates how tDCS can transiently amplify vigilance systems before cortical regulation stabilizes, if it does. Future trials should consider extracephalic cathode placement (e.g., shoulder) to reduce cranial nerve activation and improve unilateral field targeting under left DLPFC.

#### tDCS may restore balance in dysfunctional frontal–subcortical loops in youth with post-TBI depression:

Consistent with frameworks positing that depression involves disrupted top-down and bottom-up signaling[69, 70], this case demonstrated parallel behavioral and neural indicators of dysregulated fronto–brainstem dynamics. At baseline, the participant exhibited severe depressive symptoms (PHQ-9 = 22), elevated anxiety (GAD-7 = 17), and high post-concussive symptom burden (PCSS = 39), alongside selective hypoconnectivity in DLPFC–brainstem pathways (e.g., Right Area 8 → Left PRF, z = −3.60; Right Area 44→Right SNr, z = −2.00), suggesting impaired cortical modulation of subcortical arousal systems.

The behavioral changes post-tDCS coincided with correlation sign reversals in fronto–brainstem connectivity, including Right Area 9/46 → Right PRF (z = −3.01 →+2.42), Area 8 L → PAG (z = +0.40 → +2.70), and Area 9/46 L → ION R (z = −0.11 → +2.58), indicating emergent hyperconnectivity with ascending arousal nuclei. In contrast, Dorsal Area 9/46 L → RN_2 L (z = +1.69 → −3.13) worsened, and SN connectivity remained low. This circuit-specific shift supports theories of transient bottom-up re-engagement preceding restoration of top-down control [71–73]. The participant’s partial mood improvement despite worsening anxiety/arousal may reflect this transitional neural state. These findings directly justify our study aims by: (1) confirming baseline physiological disruption, (2) demonstrating tDCS-induced network change, and (3) suggesting that rs-FC correlation patterns may help identify which youth are most responsive to prefrontal tDCS.

### Occupational and Social Profile

The patient’s injuries spanned key developmental windows: *Industry vs. Inferiority* (ages ~6–11) and *Identity vs. Role Confusion* (ages ~12–18). These are periods when youth consolidate competence, social roles, and vocational identity[74]. Recurring neurological disruption during these stages may have impeded psychosocial development, contributing to identity diffusion or reduced engagement in meaningful roles [74–77].Empirical evidence supports this interpretation. A large Scottish cohort study (n > 760,000) found that children with a history of hospitalized TBI had higher odds of special educational needs (OR 1.28), more school absences/exclusions (IRR 1.09–1.33), and lower academic attainment than peers, even after adjusting for sociodemographic variables [78]. Integrating the findings of our cases study with a psychosocial developmental lens underscores that neurofunctional changes are embedded in a biopsychosocial trajectory, not merely neural connectivity metrics. Future case reports and clinical studies should include structured assessments of occupational roles, educational milestones, and identity development. This would allow researchers to map neural findings to real-world participation outcomes and better identify which youth may benefit from neuromodulatory treatment in the context of disrupted developmental continuity.

### Case Study Strengths

This case contributes preliminary evidence linking pre- and post-tDCS resting-state functional connectivity between DLPFC regions and brainstem structures in an older adolescent participant. Second, ROIs were selected a priori from arousal/mood circuitry (PAG, PRF, raphe nuclei, RN, substantia nigra). Third, circuit specificity is quantified, documenting both hypo- and hyper-connectivity and polarity flips, indicating nonuniform network responses. Finally, state-dependence is evident: PHQ-9 decreased modestly while GAD-7 and PCSS increased, suggesting different symptom domains track distinct circuits.

### Case Study Limitations

This study is limited by its small sample size, with one adolescent participant compared to ten healthy controls. While the findings are not sufficient to draw definitive conclusions, the exploration of cortical–subcortical functional connectivity before and after tDCS represents a novel line of inquiry that may guide future research. Another limitation involves sex distribution (female case vs. mixed-sex controls) also limits generalizability, warranting future studies that examine hormonal and development influences on cortical and subcortical structures.

Additionally, incomplete analysis of the median raphe outliers constrains interpretation of serotonergic network dynamics. Prior evidence suggests that TBI may selectively reduce arousal-promoting neurons in the brainstem[79],, and that tDCS can modulate these circuits through both cortical and cranial-nerve pathways [73]. In this context, several of the observed fronto–brainstem correlations (e.g., DLPFC–raphe, PRF, PAG, SN) likely reflect interactions within arousal and affective regulation systems. These limitations highlight the need to account for brainstem contributions, cranial-nerve co-stimulation, and arousal-system variability when interpreting tDCS outcomes in individuals with chronic post-concussive symptoms.

## Conclusion

This case study demonstrates that adolescent mTBI can produce lasting disruptions in cortico–brainstem communication, even years after injury. Targeted neuromodulation using left-DLPFC tDCS altered brain networks, sometimes restoring and other times reversing connectivity polarity, highlighting the sensitivity of fronto-brainstem circuits to stimulation. Clinically, mild improvement in depression with concurrent increases in anxiety and concussion symptoms suggests that modulation effects may initially heighten arousal before stabilizing control networks. Together, these findings emphasize three takeaways: (1) brainstem involvement is crucial in chronic postmTBI symptoms, (2) tDCS can dynamically reshape DLPFC–brainstem connectivity, and (3) stimulation parameters and individual neurophysiology critically influence outcomes. More broadly, these results reinforce that mTBI functions as a chronic, evolving condition rather than an acute event, with enduring network-level disturbances that warrant long-term, mechanism-informed intervention and monitoring.

## Figures and Tables

**Table 1:** Abbreviations covering clinical measures, neuroanatomy, imaging/analysis terms and procedures.

Abbrev.	Definition	Abbrev.	Definition
TBI	Traumatic Brain Injury	SN	Substantia Nigra
mTBI	mild Traumatic Brain Injury	SNr	Substantia Nigra, Pars Reticulata
PCSS	Post-Concussion Symptom Scale	SNc	Substantia Nigra, Pars Compacta
GAD-7	Generalized Anxiety Disorder–7	ION	Inferior Olivary Nucleus
PHQ-9	Patient Health Questionnaire–9	OFC	Orbitofrontal Cortex
WASI-II	Wechsler Abbreviated Scale of Intelligence–Second Edition	V1	Ophthalmic branch of trigeminal nerve
PANESS	Physical and Neurological Examination for Subtle Signs	rs-FC	resting-state Functional Connectivity
NHPT	Nine-Hole Peg Test	fMRI	functional Magnetic Resonance Imaging
DLPFC	Dorsolateral Prefrontal Cortex	MRI	Magnetic Resonance Imaging
ACC	Anterior Cingulate Cortex	BOLD	Blood-Oxygen-Level Dependent
MCC	Middle Cingulate Cortex	SENSE	Sensitivity Encoding
DMN	Default Mode Network	TR	Repetition Time
DR	Dorsal Raphe	TE	Echo Time
MR	Median Raphe	MPRAGE	Magnetization-Prepared Rapid Gradient Echo
NRM	Nucleus Raphe Magnus	tDCS	transcranial Direct Current Stimulation
PAG	Periaqueductal Gray	IRB	Institutional Review Board
PRF	Pontine Reticular Formation	ROI	Region of Interest
RN	Red Nucleus	RRC	ROI-to-ROI Connectivity
RN1	Red Nucleus subregion 1	CONN	CONN Functional Connectivity Toolbox
RN2	Red Nucleus subregion 2	BIDS	Brain Imaging Data Structure
Eq.	Equation	SD	Standard Deviation
z (z-score)	Standard score	IQR	Interquartile Range

**Table 2:** Pre- and post-intervention behavioral scores for the TBI participant alongside reference sample means. Standard deviations (SD).

Measure	Pre	Post	Change	Reference Mean (SD)
Age (years)	19	19	0	17.8 (3.55)
PCSS	39	46	+7	4.9 (6.08)
GAD-7	17	20	+3	3.5 (3.81)
PHQ-9	22	19	−3	4.2 (2.82)
WASI-II Matrix Reasoning	16	16	0	15.6 (4.62)
WASI-II Vocabulary	34	41	+7	38.4 (4.88)

**Table 2:** Pre- and post-stimulation z-scores for dorsolateral prefrontal–brainstem connectivity across homologous hemispheric pairs. Cell colors reflect the relative magnitude and direction of Fisher z–transformed connectivity values, with green indicating stronger positive deviations, red indicating stronger negative deviations, and yellow representing values near the group mean (z ~ 0) for each DLPFC–brainstem connection at pre- and post-tDCS timepoints

Pre-Frontal ROIs?	Brainstem ROIs	Pre Z-Scores	Post Z-scores (Outliers=0)	Pre-Frontal ROIs	Brainstem ROIs	Pre Z-scores	Post Z-scores (Outliers=0)
Area8_Right	DR	0.456	−1.736	Area8_Left	DR	1.236	0.880
Area8_Right	ION_Left	−1.530	−0.226	Area8_Left	ION_Left	−1.575	0.231
Area8_Right	ION_Right	−0.508	−0.241	Area8_Left	ION_Right	−0.109	2.584
Area8_Right	MR	−0.593	0.000	Area8_Left	MR	0.250	0.000
Area8_Right	PAG	−0.343	0.715	Area8_Left	PAG	0.395	2.701
Area8_Right	PRF_Left	−3.603	−2.238	Area8_Left	PRF_Left	0.139	0.868
Area8_Right	PRF_Right	−0.018	0.913	Area8_Left	PRF_Right	−1.211	1.992
Area8_Right	NRM	−0.378	−0.605	Area8_Left	NRM	−1.533	1.665
Area8_Right	RN1_Left	−0.467	−1.528	Area8_Left	RN1_Left	0.681	−0.084
Area8_Right	RN1_Right	1.555	0.478	Area8_Left	RN1_Right	−0.309	−0.833
Area8_Right	RN2_Left	−0.092	−0.184	Area8_Left	RN2_Left	0.188	−0.453
Area8_Right	RN2_Right	−0.117	−0.789	Area8_Left	RN2_Right	−0.100	−0.530
Area8_Right	SNr_Left	−0.149	−0.623	Area8_Left	SNr_Left	0.556	−0.361
Area8_Right	SNr_Right	0.772	0.928	Area8_Left	SNr_Right	1.691	−1.435
Area8_Right	SNc_Left	0.865	−0.336	Area8_Left	SNc _Left	0.118	−0.430
Area8_Right	SNc_Right	1.628	1.848	Area8_Left	SNc_Right	0.880	−0.898
Area9/46_Right	DR	0.755	−1.580	Area9/46_Left	DR	1.331	−0.757
Area9/46_Right	ION_Left	1.058	−0.784	Area9/46_Left	ION_Left	1.708	1.397
Area9/46_Right	ION_Right	1.167	1.087	Area9/46_Left	ION_Right	0.030	1.001
Area9/46_Right	MR	−0.586	0.000	Area9/46_Left	MR	0.373	0.000
Area9/46_Right	PAG	−0.378	−1.218	Area9/46_Left	PAG	0.132	0.440
Area9/46_Right	PRF_Left	0.869	0.629	Area9/46_Left	PRF_Left	0.290	0.932
Area9/46_Right	PRF_Right	−3.013	2.419	Area9/46_Left	PRF_Right	0.378	0.965
Area9/46_Right	NRM	0.218	−0.702	Area9/46_Left	NRM	1.687	0.034
Area9/46_Right	RN1_Left	0.250	0.102	Area9/46_Left	RN1_Left	0.505	0.260
Area9/46_Right	RN1_Right	0.135	−1.021	Area9/46_Left	RN1_Right	−0.279	0.213
Area9/46_Right	RN2_Left	1.579	−0.804	Area9/46_Left	RN2_Left	1.694	−3.131
Area9/46_Right	RN2_Right	1.181	1.264	Area9/46_Left	RN2_Right	0.282	−0.260
Area9/46_Right	SNr_Left	−0.158	−0.093	Area9/46_Left	SNr_Left	1.154	−1.025
Area9/46_Right	SNr_Right	−0.087	0.429	Area9/46_Left	SNr_Right	−1.285	−0.237
Area9/46_Right	SNc_Left	0.313	1.446	Area9/46_Left	SNc_Left	−0.540	−1.011
Area9/46_Right	SNc_Right	−0.003	0.859	Area9/46_Left	SNc_Right	−1.547	−0.611
Area44_Right	DR	−0.828	0.828	Area44_Left	DR	−0.854	0.401
Area44_Right	ION_Left	0.781	−1.263	Area44_Left	ION_Left	−0.566	1.566
Area44_Right	ION_Right	1.534	−0.277	Area44_Left	ION_Right	−0.650	0.637
Area44_Right	MR	−0.663	0.000	Area44_Left	MR	0.983	0.000
Area44_Right	PAG	−0.355	1.695	Area44_Left	PAG	−0.835	0.107
Area44_Right	PRF_Left	−0.926	−3.885	Area44_Left	PRF_Left	−0.373	0.118
Area44_Right	PRF_Right	0.337	−1.173	Area44_Left	PRF_Right	0.029	−0.642
Area44_Right	NRM	−1.424	1.180	Area44_Left	NRM	−0.448	−0.182
Area44_Right	RN1_Left	−1.373	0.043	Area44_Left	RN1_Left	−2.034	1.507
Area44_Right	RN1_Right	0.709	0.780	Area44_Left	RN1_Right	−0.558	0.863
Area44_Right	RN2_Left	−1.418	0.958	Area44_Left	RN2_Left	−0.629	−1.180
Area44_Right	RN2_Right	1.525	1.044	Area44_Left	RN2_Right	−0.639	−0.240
Area44_Right	SNr_Left	−2.159	−0.102	Area44_Left	SNr_Left	−0.230	−0.001
Area44_Right	SNr_Right	−2.044	−2.244	Area44_Left	SNr_Right	−2.105	0.798
Area44_Right	SNc_Left	0.278	0.281	Area44_Left	SNc_Left	0.601	0.188
Area44_Right	SNc_Right	0.054	−1.966	Area44_Left	SNc_Right	−0.095	−0.521

## Data Availability

The datasets generated during and/or analyzed during the current study are not publicly available due to sensitivity of data and ongoing data collection, but are available from the corresponding author on reasonable request.
